# Dietary Fructose and the Metabolic Syndrome

**DOI:** 10.3390/nu11091987

**Published:** 2019-08-22

**Authors:** Marja-Riitta Taskinen, Chris J Packard, Jan Borén

**Affiliations:** 1 Research Program for Clinical and Molecular Medicine Unit, Diabetes and Obesity, University of Helsinki, 00029 Helsinki, Finland; 2 Institute of Cardiovascular and Medical Sciences, University of Glasgow, Glasgow G12 8QQ, UK; 3 Department of Molecular and Clinical Medicine, University of Gothenburg and Sahlgrenska University Hospital, 41345 Gothenburg, Sweden

**Keywords:** fructose, metabolic syndrome, hypertriglyceridemia, metabolism

## Abstract

Consumption of fructose, the sweetest of all naturally occurring carbohydrates, has increased dramatically in the last 40 years and is today commonly used commercially in soft drinks, juice, and baked goods. These products comprise a large proportion of the modern diet, in particular in children, adolescents, and young adults. A large body of evidence associate consumption of fructose and other sugar-sweetened beverages with insulin resistance, intrahepatic lipid accumulation, and hypertriglyceridemia. In the long term, these risk factors may contribute to the development of type 2 diabetes and cardiovascular diseases. Fructose is absorbed in the small intestine and metabolized in the liver where it stimulates fructolysis, glycolysis, lipogenesis, and glucose production. This may result in hypertriglyceridemia and fatty liver. Therefore, understanding the mechanisms underlying intestinal and hepatic fructose metabolism is important. Here we review recent evidence linking excessive fructose consumption to health risk markers and development of components of the Metabolic Syndrome.

## 1. Introduction

Food patterns and diet have greatly changed during the last decades in both industrialized and developing countries together with sedentary lifestyle resulting in dramatic increases of obesity, Metabolic Syndrome (MetS), non-alcoholic fatty liver disease (NAFLD), and type 2 diabetes [1,2,3,4]. Importantly, the rapid increase in pediatric NAFLD has become the major concern globally [5,6]. As obesity is a driving force for NAFLD and type 2 diabetes, it is not surprising that the prevalence of the MetS is high in both disorders.

The main component of dietary changes is not only lack of physical activity in face of extra calories, but in particular increases of added sugar mainly in sugar sweetened beverages (SSBs). The common sweeteners are sucrose (containing 50% saccharose and 50% fructose) and high fructose corn syrup (containing up to 55% fructose). The consumption of SSBs, comprising fruit-flavored drinks and sport and energy drinks, is the main source of added sugar. It accounts for about 15–17% of the total daily energy intake in Western diets. Thus, it exceeds the recommended limit of 5% of added sugar (World Health Organization’s guidelines 2018) [7]. Consequently, excess sugar consumption has become a major public health problem particularly in children and teen-age populations globally. This menace has initiated the call for the restriction of sugar consumptions [8].

There is substantial and consistent data evidence that exposure to excess fructose intake has detrimental effects on multiple cardiometabolic risk factors [9,10,11,12,13]. In fact, fructose consumption is considered to be a culprit in the MetS as a lipogenic compound that associates with excess ectopic fat accumulation, particularly in the liver. This review will focus on the links between fructose consumption and the MetS, highlighting specifically effects of fructose on hepatic lipid homeostasis and metabolism.

## 2. Metabolic Effects of Fructose Consumption

### 2.1. Fructose Metabolism in Enterocytes

**Figure 1:**
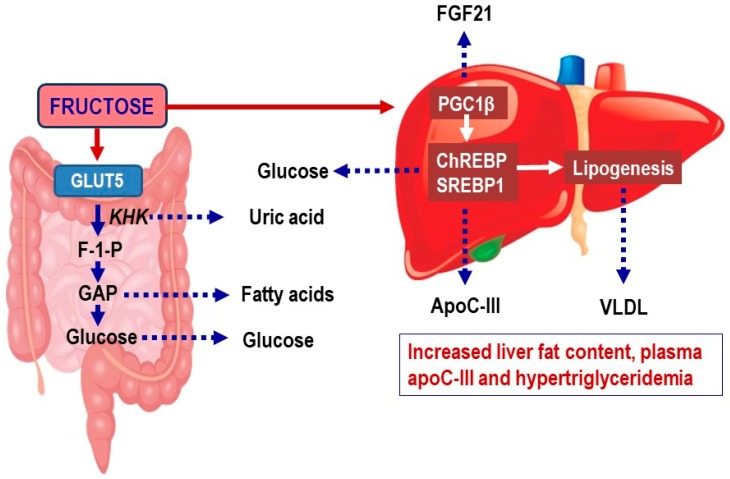
Metabolism of fructose in the intestine and liver. Fructose is in the small intestine metabolized by ketohexokinase (KHK) into fructose-1-phosphate (F-1-P) [19]. F-1-P is then cleaved by aldolase B into dihydroxyacetone phosphate and glyceraldehyde. Glyceraldehyde is phosphorylated by triokinase generating glyceraldehyde 3-phosphate (GAP). GAP and other triose phosphates are resynthesized into glucose via gluconeogenesis or metabolized into lactate or acetyl-CoA, which are oxidized or used for lipogenesis. In the liver, fructose activates the transcription factors carbohydrate-responsive element-binding protein (ChREBP) and sterol regulatory element-binding transcription factor 1c (SREBP1c) and their coactivator peroxisome proliferator-activated receptor-β (PGC1β) [16]. This results in upregulation of pathways that stimulate fructolysis, glycolysis, lipogenesis, and glucose production. Collectively, this results in increased hepatic glucose production, generation of lipid intermediates that may affect hepatic insulin sensitivity, increased expression of APOC3 and increased secretion of triglyceride-rich very-low density lipoproteins (VLDL). The increased APOC3 expression induces increased plasma apoC-III, an inhibitor of lipoprotein lipase and hepatic clearance of lipoprotein remnants [20]. This results in hypertriglyceridemia and accumulation of atherogenic triglyceride-rich lipoprotein (TRL) remnants.

Although fructose and glucose are both monosaccharides with closely similar formulas, their metabolism pathways are divergent in both enterocytes and in hepatocytes [14,15,16,17]. Fructose absorption is mainly mediated by glucose transporter 5 (GLUT-5), a fructose transporter expressed on the apical border of enterocytes in the small intestine across the lumen in enterocytes (Figure 1) [17,18]. Fructose trafficking from the enterocytes into the portal vein is partly also mediated by GLUT-2. Notably, a part of fructose is metabolized in the cytosol by fructokinase, an enzyme that catalyzes the transfer of a high-energy phosphate group to d-fructose. Notably, high flux of fructose into enterocytes induces GLUT-5 expression. This mechanism may respond to excess chronic fructose intake by increasing the capacity of the intestine for fructose absorption and transport to the liver. Thus, GLUT-5 activity is the key regulator of fructose concentration in the portal vein.

Carbohydrate response element binding protein (ChREBP), a transcription factor responding to sugar intake, is recognized to be a key regulator of hepatic carbohydrate and lipid metabolism [21,22]. Recent data highlight the role of ChREBP in enterocytes as a regulator of intestinal GLUT-5 expression [19,23]. Interestingly, chronic fructose feeding in hamsters enhances lipid synthesis in enterocytes, resulting in increased synthesis of apoB48 and release of intestinal-derived chylomicrons, likely by activation of ChREBP and GLUT-5 [24]. In contrast, ChREBP-deficient mice fed a high fructose diet are reported to be fructose intolerant due to impaired fructose absorption and decreased expression of GLUT-5 [19]. These data highlight the role of intestinal ChREBP for fructose-induced impaired metabolism.

The liver is considered to be the major organ for fructose metabolism [16,25,26,27]. Plasma concentration of fructose are increased only trivially after fructose intake in humans as first pass metabolism by liver covers about 80–90% of the fructose load. Recently, this concept has been challenged by studies in mice utilizing isotope tracers and mass spectrometry [28]. The key finding suggests that the small intestine may be the major site for dietary fructose metabolism instead of the liver. Notably, the intestinal fructose metabolism seems to be a saturable process that allows high doses of dietary fructose to pass to the liver. The handling of dietary fructose in humans may be different due to the relatively smaller gut in humans than in mice [29]. This is supported by studies in healthy volunteers with stable isotope-labelled fructose to study the initial metabolism of ingested fructose [30]. The amount of dietary fructose escaping the splanchnic extraction averaged only about 14.5% and the first-pass extraction was 85.5%.

A study in healthy males (*n* = 7) demonstrated that fructose combined with Intralipid infusion (consisting of 10% soybean oil, 1.2% egg yolk phospholipids, 2.25% glycerin, and water) resulted in increased apoB48 production rate without any altered catabolism of chylomicrons [31]. Recently, we reported that in abdominally obese men, fructose consumption (75 g/per day served as fructose sweetened beverage) for 12 weeks increased postprandial responses of both plasma triglycerides and apoB48 to a fat rich meal [32]. How changes in fructose absorption and metabolism in enterocyte influence intestinal lipogenesis, reflected in handling of dietary fats and postprandial lipemia, remains to be established in future kinetic studies in humans as direct extrapolation from animal studies may be misleading.

### 2.2. Fructose Metabolism in the Liver

Hepatic fructose and glucose metabolism occurs via divergent pathways with consequences on hepatic lipid handling and insulin sensitivity reflected in metabolic diseases [15,27,33]. Fructose uptake from portal circulation into liver is mediated by GLUT2 via first pass metabolism by phosphorylation of fructose to fructose-1-phosphate, that is further metabolized to dihydroxyacetone phosphate and glyceraldehyde 3-phosphate. Notably only a small part of ingested fructose ends up in the circulation in contrast to glucose. These initial steps in fructose metabolism seem to be unregulated and bypass the hormonal control in contrast to the strictly regulated glucose uptake and glycolysis in the liver where insulin plays a central role. Glyceraldehyde 3-phosphate and other triose phosphate compounds derived from fructolysis are directed to the formation of pyruvate and acetyl-CoA and to lipogenesis. Consequently, fructose has effects on both glucose homeostasis and lipogenesis the partitioning depending on cellular energy needs [34].

### 2.3. Evidence Linking Fructose Intake to Non-Alcoholic Fatty Liver Disease (NAFLD) and to Increased Cardiometabolic Risk

The heterogeneity of obesity and its consequences on cardiometabolic risk has been addressed in several outstanding recent reviews that have recognized the importance of body fat distribution, in particular the ectopic fat in the liver as the critical link to cardiometabolic health [25,35,36,37,38,39,40]. The central role of non-alcoholic fatty liver disease (NAFLD) as the source for multiple cardiometabolic risk factors has raised the questions how the liver can handle extra influx of lipids and the consequences on lipoprotein metabolism and ultimately vascular health [9,10,13,41].

### 2.4. Effects of Fructose on Hepatic De Novo Lipogenesis

The hallmark of NAFLD is hepatic triglyceride accumulation. The disease develops when the influx of lipid into the liver (from circulating non-esterified fatty acids, diet-derived chylomicrons, and hepatic *de novo* lipogenesis(DNL)) exceeds hepatic lipid disposal (via β-oxidation in mitochondria and triglyceride secretion as lipoprotein particles) [42]. In the past decade, we have seen a remarkable increase in NAFLD [43,44,45]. It is already the most common cause of chronic liver disease in Western countries and may soon achieve this status in the rest of the world.

Accumulating evidence indicates that increased hepatic DNL is a significant pathway contributing to the development of NAFLD [6,46]. Dietary carbohydrates, in particular, fructose, have been shown to stimulate DNL and increase liver fat, although it is still debated whether this is due to excess energy or fructose per se [46]. Studies in humans are lacking but a comparison between fructose and glucose supplementation in rats for two months showed that, although total caloric consumption was higher in glucose-supplemented rats, fructose caused worse metabolic responses [47].

DNL is a highly regulated pathway, dependent upon several steps, in which key enzymes involved are upregulated in NAFLD [48,49,50]. Importantly, dietary fructose further increases levels of enzymes involved in DNL as fructose is absorbed via portal vein and delivered to the liver in much higher concentrations as compared to other tissues. Interestingly, in contrast to metabolism of glucose, the breakdown of fructose leads to the generation of metabolites that stimulate hepatic DNL [51,52,53].

Fructose drives lipogenesis in the setting of insulin resistance as fructose does not require insulin for its metabolism, and it directly stimulates sterol regulatory element-binding protein 1 (SREBP-1c), a major transcriptional regulator of DNL (Figure 1) [54]. Fructose also promotes hepatic DNL and lipid accumulation by suppressing hepatic β-oxidation and by inducing promotes ER stress and uric acid formation (see below). High-fructose feeding has also been shown to increase hepatic expression of ChREBP, a lipogenic transcription factor of carbohydrate metabolism and DNL. ChREBP regulates fructose-induced glucose production independently of insulin signaling [22], and the fructose-induced increases in circulating fibroblast growth factor 21 (FGF21) [55]. The fructose-induced FGF21 feeds back on the liver to enhance further ChREBP activity and hepatic DNL and VLDL secretion [55]. Consequently, circulating FGF21 levels correlate with rates of de novo lipogenesis in human subjects [55]. FGF21 has also been implemented in a signaling axis regulating carbohydrate consumption [11,16,27,33,46,56].

Despite convincing evidence in animals, whether fructose consumption increases DNL in humans to the extent that it induces metabolic disturbances has been more controversial [57]. However, Stanhope et al. reported that 10 weeks overfeeding of fructose (but not glucose), increased DNL (from 11% to 17%) [58]. Other studies have reported that a high fructose diet increased fasting DNL from 2% to 9% [59], and that fructose (75 g/day), served with their habitual diet over 12 weeks to abdominally obese men resulted in significant increases in DNL in both the fasting state (12.3% to 16.5%) and 4 to 8 h postprandially [32,60]. In line, 9 days of isocaloric fructose restriction in the context of an otherwise normal diet led to significant decreased DNL in 37 out of 40 children with obesity [61].

### 2.5. Clinical Evidence That Fructose Consumption Is Leading to Non-Alcoholic Fatty Liver Disease (NAFLD)

As fructose is recognized to be a lipogenic sugar, its contribution to the pathogenesis of NAFLD has been the focus of intensive research for more than a decade [16,27,33,54,58,62]. The ongoing interest is stimulated by the huge global burden of NAFLD as the potential driver of CVD and its clinical manifestations [11,15,16,63,64,65].

Although accumulated evidence has demonstrated a strong link between fructose consumption and NAFLD it is still unclear if the association is caused by fructose consumption per se, or by the increased energy intake [66]. An important reason for this is the technological problems associated with measuring liver fat content. Accurate non-invasively measurements of liver fat content require advanced equipment like magnetic resonance imaging and spectroscopy [66,67,68,69]. Notably, these technologies allow both quantitation and characterization of hepatic lipids [69,70]. NAFLD is commonly define as >5.5% hepatic fat fraction as determined by MRI [71].

Mot studies focusing on the association between fructose or saccharose overfeeding and liver fat steatosis (quantitated by MRI), have been performed in healthy or obese men [58,72,73,74,75,76,77,78,79]. Many of these studies have been positive [58,72,73,74,75,76,77,78,79]. For example, daily intake of one liter regular cola for 6 months in overweight subjects (*n* = 10) was shown to associate with significant increases of liver fat measured by MRI, but without significant changes of BMI or total fat mass [80]. However, many studies have also been negative [81,82,83].

Reasons for the different outcome from these earlier studies are the relatively smaller study cohorts, variable and short-duration less than 7 days) study designs, and differing doses of fructose. Despite these weaknesses, many studies seem to indicate that hypercaloric fructose feeding increases liver fat content and that this response is aggravated in obese subjects. For example, Ma et al. reported that the regular consumption of SSBs in overweight and obese subjects from the Framingham cohort (*n* = 2634) was associated with increases of liver fat content quantitated by computed tomography [84].

In line, we recently reported that fructose consumption (75 g/day as fructose sweetened beverage) for 12 weeks in abdominally obese men with cardiometabolic risk factors, significantly increased liver fat content measured by MRI despite relative low increases in weight and waist circumferences [32]. The study subjects were served their habitual diet with add on fructose feeding resulting in hypercaloric set up that also occurs in the real world with SSB intake [32].

Despite this study design, the average increase of liver fat content was modest (10%) in face of no significant change in visceral or subcutaneous fat depots and there was high variation in the response liver fat content. To better understand this variation, we genotyped all individuals for carrier status of the major risk alleles for hepatic steatosis; PNPLA3, TN6SF2, and MBOAT7. Results showed that the number of risk alleles associated with increased liver fat at the baseline. However, individuals without and with risk allele did not have differences in the response of liver fat during fructose feeding. In line with this, two other studies have confirmed increases of liver fat content during carbohydrate (simple sugars) overfeeding on hypercaloric diet for 3 weeks in overweight and obese subjects [85,86].

The key metabolic and mechanistic issues of fructose consumption has been studied during a nine days isocaloric feeding study (fructose restriction to less than 4% of calories) in obese children (*n* = 40) with MetS and a high habitual sugar consumption (>50 g/day) [61,87,88]. The metabolic assessment was extensive utilizing magnetic resonance imaging and stable isotope technology for DNL in addition to extensive biomarker platform. The first important message is that the dietary fructose restriction was associated with significant reductions of liver (from 7.2% to 3.8%, *p* < 0.001) and visceral fat content (from 123 cm^3^ to 110 cm^3^, *p* < 0.001). Notably, the reduction of liver fat content was not related to the baseline liver fat content. The diet intervention was also associated with a significant decrease of DNL and an improved lipoprotein profile. In addition, significant improvements were observed in biomarkers of insulin resistance and glucose metabolism. The authors also elucidated the impact of the diet intervention on the methylglyoxal (MG) pathway [15,63,89,90,91], and surprisingly found that fructose restriction associated with marked reduction of D-lactate, a biomarker of MG metabolism. This change of D-lactate correlated with reduction of liver fat content and DNL [88]. These observations open a new perspective of the adverse metabolic effects of excess fructose intake.

Thus, accumulating evidence supports the fact that fructose is an important mediator for the development of NAFLD and a main driver for DNL [64]. Indeed, this concept is supported by a recent meta-analysis including 6326 participants and 1361 cases with NAFLD [92]. However, it is still debated whether fructose, when consumed in isocaloric amounts, causes more liver fat accumulation than other energy-dense nutrients [93].

Kirk et al. investigated the effects of acute and chronic calorie restriction with either a low-fat, high-carbohydrate (>180 g/day) diet or a low-carbohydrate (<50 g/day) diet on hepatic and skeletal muscle insulin sensitivity in 22 obese subjects [94]. Interestingly, the low-carbohydrate diet lowered the hepatic lipids within 48 h by 30% (compared to ~10% in the low-fat, high-carbohydrate group). The mechanism for the rapid clearance of liver fat was not elucidated. After approximately 11 weeks (7% weight loss) a similar marked reduction of liver fat content was seen in both groups (38% vs. 44%). These results show that liver fat content is highly dynamic in response to energy balance and sugar intake. However, it is still debated whether a low-carbohydrate hypocaloric diet is more efficient than a low-fat hypocaloric diet in reducing intrahepatic lipid accumulation.

Haufe et al. compared the 6 months responses of a low-carbohydrate hypocaloric or a low-fat hypocaloric diet on intrahepatic lipid accumulation in overweight/obese subjects (*n* = 84 to 86 in each group) [95]. Results showed that both diets had the same beneficial effects on intrahepatic lipid accumulation, weight loss and insulin resistance. The decrease in intrahepatic fat was independent of visceral fat loss and not associated with changes in whole body insulin sensitivity. Interestingly, subjects with high baseline intrahepatic lipids (>5.6%) lost ≈ 7-fold more liver fat compared with those with low baseline values irrespective of the prolonged hypocaloric diet.

### 2.6. Effects of Fructose on Uric Acid Metabolism and MG (Methyl Glyoxal) Pathways

The role of fructose as a potential source of uric acid was recognized decades ago [96]. The rapid phosphorylation of fructose to fructose-1-phospate not only increases the fluxes of trioses for lipogenesis, but also depletes ATP stores leading to the degradation of AMP, resulting in increased generation of uric acid via purine pathway. Importantly, fructose seems to be the only carbohydrate that can generate uric acid. Cellular depletion of ATP has several adverse consequences on energy metabolism including increased ER stress and mitochondrial dysfunction [64]. ER stress has been linked to many metabolic diseases including NAFLD [97] and can be induced by a range of condition such as high protein demand, viral infection, mutant protein expression, hypoxia, energy deprivation, or exposure to excessive oxidative stress including ATP depletion [97,98,99]. The ER stress triggers an adaptive signaling pathway known as the Unfolded Protein Response (UPR) to restore normal ER function. If the UPR fail to restore normal ER function, the UPR aims towards apoptosis [97,98,99]. A sustained chronic UPR response may worsen the pathophysiological condition by inducing lipotoxicity, insulin resistance, inflammation, and apoptotic cell death [97,98,99].

ER stress activates the transcription factor X-box binding protein 1 (XBP1s), a key regulator of the unfolded protein response. Interestingly, XBP1 also regulates hepatic fatty acid synthesis [100]. Mitochondrial oxidative stress results in enhanced generation of citrate and acetyl coenzyme A AcCoA [101,102,103], two metabolites that stimulate lipogenesis. This may explain why fructose is lipogenic [16,104]. An additional nexus is that increased triose flux enhance the generation of methyl glyoxal (MG) and dicarbonyl stress. A key crossroad step is the inactivation of AMPK by MG and consequences on energy metabolism. This is a novel pathway linked to excess fructose intake and its metabolic relevance remains to be clarified [15]. In summary, fructose seems to influence multiple metabolic pathways in the liver that results in enhanced lipogenesis, generation of uric acid, ER stress, and inflammation. The association between uric acid and insulin resistance [105], raised the interest of uric acid as a potential biomarker in the Metabolic Syndrome [105]. Indeed, several studies have established that serum uric acid is a risk factor for the Metabolic Syndrome [106,107,108,109,110,111,112,113].

Substantial evidence suggest that uric acid also associates with NAFLD [114,115,116,117]. Several studies have reported that serum uric acid levels are higher in subjects with NAFLD than in those without NAFLD [64,118,119]. Importantly, hyperuricemia seems to associate with NAFLD independently of other features of the MetS and these associations are independent on body weight [120]. Thus, uric acid belongs to the cluster of biomarkers in Metabolic Syndrome and NAFLD.

Can excess intake of fructose and SSBs result in the elevation of plasma uric acid concentrations? Indeed, numerous studies have found an association between fructose/SSBs intake and uric acid levels [27,54,64,118,121,122]. For example, two large prospective cohort studies including American men (*n* = 46,393) or American women (*n* = 78,906) showed strong associations between consumption of SBBs and hyperuricemia [123,124]. Similar positive associations between SSBs intake and serum uric acid concentrations have been observed in Korean, Mexican, and Brazilian populations [125,126,127]. Likewise, in an adolescents cohort (*n* = 4867 aged 12 to 18 years) consumption of SSBs associated with higher serum uric acid and also higher systolic blood pressure [128].

So far, data from RCTs on fructose feeding trials have remained limited. Fructose feeding associated with increased uric acid in three smaller intervention studies [72,129,130]. Weaknesses of the study protocol are that the design and duration of feeding trials, as well as study cohorts, are highly variable. Unfortunately, data from available meta-analyses are not consistent. One meta-analysis reported that fructose intake as an apart of isocaloric diet did not raise uric acid levels but signaled that the hypercaloric intake of fructose may raise uric acid [131]. Although hyperuricemia is highly prevalent in patients with NAFLD and Met Syndrome, its clinical relevance remains debated. Recent French recommendations for sugar intake concluded that long-term consequences of potential small increases of uric acid by fructose/sugar intake remain insufficient [132], likewise critical analysis of the available data left open the casual link between fructose intake and hyperuricemia [133,134].

### 2.7. Consequences of Increased Lipid Synthesis to Very Low-Density Lipoproteins (VLDL) Metabolism and Release—Effects on Plasma Lipids, Lipoproteins, and Apolipoproteins

The association of blood lipid levels and consumption of added sugars was studied in the adult population in the National Health and Nutrition Examination Survey (NHANES) (*n* = 6113) [135]. In this American cross-sectional study higher fructose consumers had more unfavorable lipid levels, namely significantly lower HDL cholesterol, higher triglycerides, and a high ratio of triglycerides to high density lipoproteins (HDL), whereas women also had higher low-density lipoprotein (LDL) cholesterol levels. Likewise, in the Framingham study, daily soft drink consumers had higher incidence of elevated triglycerides and low HDL cholesterol than non-consumers (relative risk: 1.22 and 1.22, respectively) [136].

Several studies have consistently reported increased responses of fasting and postprandial triglyceride levels and 24 h. profiles to short term feeding of fructose as compared to glucose in both lean and obese subjects [8,53]. These perturbations directly lead to other lipid abnormalities including elevation of apoB levels, accumulation of small dense LDL, and increased remnant lipoproteins, combined with reduced HDL cholesterol which all are components of the atherogenic lipid triad, a strong risk factor for CVD. Interestingly, the deleterious effect of fructose on lipid metabolism is directly linked to the daily intake; a fructose intake >50 g/day is associated with postprandial hyperlipemia whereas intake above 100 g/day also results in elevation of fasting serum triglycerides [137]. Collectively, these results clearly show that fructose intake is directly linked to an atherogenic dyslipidemia.

The recent increased focus on plasma triglycerides and postprandial hyperlipidemia not only as markers but also as causal drivers of CVD has partly been driven by improved understanding of the biology and genetics of triglyceride heritability. Of particular interest is *APOC3*, which has emerged as a novel therapeutic target to reduce dyslipidemia and CVD risk [20,138,139,140]. Interestingly, fructose feeding is linked to a significant rise of plasma apoC-III levels (Figure 1) [32,141].

### 2.8. Interactions between Fructose Consumption and Changes in Gut Microbiota

High consumption of fructose, artificial sweeteners, and sugar alcohols have been shown to affect host-gastrointestinal microbe interactions and possibly contribute to the development of metabolic disorders and obesity. Multiple studies have also reported fructose as a critical factor contributing to NAFLD progression by modulating intestinal microbiota (see review [142]). Gut microbiota interacts with its host, and influences both the energy homeostasis and the immunity of the host [142]. Shifts in this composition can result in alterations of the symbiotic relationship, which can promote metabolic diseases [143]. Indeed, the microbial composition have been shown to differ between healthy individuals and NAFLD patients [144], and a diet enriched in fructose not only induced NAFLD but also negatively affected the gut barrier and the microbiota composition, leading to impaired microbiota [145]. The underlying mechanisms are complex and still unclear, but Oh et al. recently showed that dietary fructose activates the Ack-pathway, involved in generating acetic acid, which in turn triggers the bacterial stress response that promotes phage production [146]. Thus, prophages in a gut symbiont can be induced by diet and metabolites affected by diet, which provides a potential mechanistic explanation for the effects of diet on the intestinal phage community [147].

The complex interaction between dietary carbohydrates and gut microbiota was recently demonstrated in a two-week intervention with an isocaloric low-carbohydrate diet in obese subjects with NAFLD [148]. The authors observed rapid and marked reductions of liver fat paralleled by marked decreases in hepatic DNL and increases in hepatic β-oxidation. Interestingly, the marked reduction in cardiometabolic risk factors paralleled with rapid increases in the folate-producing gut microbiota Streptococcus, serum folate concentrations, and hepatic one-carbon metabolism.

## 3. Conclusions

Consistent data evidence that excess fructose intake as a central component of unhealthy lifestyle has detrimental effects on multiple cardiovascular risk factors. Consequently, it is not surprising that links between fructose consumption with MetS and NAFLD are strong. Fructose is a lipogenic sugar as it increases hepatic *de novo* lipogenesis in the liver through several metabolic pathways resulting in a vicious circle that further aggravates DNL. Increased DNL favors excess fat accumulation in the liver, being a driving force for increased secretion of VLDL particles leading to the atherogenic lipid profile and other metabolic derangements associated with CVD risk. The global health burden of MetS together with NAFLD is growing rapidly, sweeping across the world. It is clear that added sugars have become a threat to cardiometabolic health. These facts call for the restriction of dietary sugars, especially SSB consumption to limit fructose intake to achieve better cardiometabolic health.

## References

[1] Afshin A., Forouzanfar M.H., Reitsma M.B., Sur P., Estep K., Lee A., Marczak L., Mokdad A.H., Moradi-Lakeh M., GBD 2015 Obesity Collaborators (2017). Health Effects of Overweight and Obesity in 195 Countries over 25 Years. N. Engl. J. Med..

[2] Bluher M. (2019). Obesity: Global epidemiology and pathogenesis. Nat. Rev. Endocrinol..

[3] Malik V.S., Li Y., Pan A., De Koning L., Schernhammer E., Willett W.C., Hu F.B. (2019). Long-Term Consumption of Sugar-Sweetened and Artificially Sweetened Beverages and Risk of Mortality in US Adults. Circulation.

[4] Younossi Z.M. (2019). Non-alcoholic fatty liver disease—A global public health perspective. J. Hepatol..

[5] Vos M.B., Abrams S.H., Barlow S.E., Caprio S., Daniels S.R., Kohli R., Mouzaki M., Sathya P., Schwimmer J.B., Sundaram S.S. (2017). NASPGHAN Clinical Practice Guideline for the Diagnosis and Treatment of Nonalcoholic Fatty Liver Disease in Children: Recommendations from the Expert Committee on NAFLD (ECON) and the North American Society of Pediatric Gastroenterology, Hepatology and Nutrition (NASPGHAN). J. Pediatr. Gastroenterol. Nutr..

[6] Younossi Z., Tacke F., Arrese M., Chander Sharma B., Mostafa I., Bugianesi E., Wai-Sun Wong V., Yilmaz Y., George J., Fan J. (2019). Global Perspectives on Nonalcoholic Fatty Liver Disease and Nonalcoholic Steatohepatitis. Hepatology.

[7] Powell E.S., Smith-Taillie L.P., Popkin B.M. (2016). Added Sugars Intake Across the Distribution of US Children and Adult Consumers: 1977–2012. J. Acad. Nutr. Diet..

[8] Johnson R.K., Lichtenstein A.H., Anderson C.A.M., Carson J.A., Despres J.P., Hu F.B., Kris-Etherton P.M., Otten J.J., Towfighi A., Wylie-Rosett J. (2018). Low-Calorie Sweetened Beverages and Cardiometabolic Health: A Science Advisory From the American Heart Association. Circulation.

[9] Lim S., Taskinen M.R., Boren J. (2019). Crosstalk between nonalcoholic fatty liver disease and cardiometabolic syndrome. Obes. Rev..

[10] Santos R.D., Valenti L., Romeo S. (2019). Does nonalcoholic fatty liver disease cause cardiovascular disease? Current knowledge and gaps. Atherosclerosis.

[11] Mirtschink P., Jang C., Arany Z., Krek W. (2018). Fructose metabolism, cardiometabolic risk, and the epidemic of coronary artery disease. Eur. Heart J..

[12] Stanhope K.L., Goran M.I., Bosy-Westphal A., King J.C., Schmidt L.A., Schwarz J.M., Stice E., Sylvetsky A.C., Turnbaugh P.J., Bray G.A. (2018). Pathways and mechanisms linking dietary components to cardiometabolic disease: Thinking beyond calories. Obes. Rev..

[13] Stahl E.P., Dhindsa D.S., Lee S.K., Sandesara P.B., Chalasani N.P., Sperling L.S. (2019). Nonalcoholic Fatty Liver Disease and the Heart: JACC State-of-the-Art Review. J. Am. Coll. Cardiol..

[14] Ferraris R.P., Choe J.Y., Patel C.R. (2018). Intestinal Absorption of Fructose. Annu. Rev. Nutr..

[15] Mortera R.R., Bains Y., Gugliucci A. (2019). Fructose at the crossroads of the metabolic syndrome and obesity epidemics. Front. Biosci. (Landmark Ed.).

[16] Hannou S.A., Haslam D.E., McKeown N.M., Herman M.A. (2018). Fructose metabolism and metabolic disease. J. Clin. Investig..

[17] Hoffman S., Alvares D., Adeli K. (2019). Intestinal lipogenesis: How carbs turn on triglyceride production in the gut. Curr. Opin. Clin. Nutr. Metab. Care.

[18] Patel C., Douard V., Yu S., Gao N., Ferraris R.P. (2015). Transport, metabolism, and endosomal trafficking-dependent regulation of intestinal fructose absorption. FASEB J..

[19] Lee H.J., Cha J.Y. (2018). Recent insights into the role of ChREBP in intestinal fructose absorption and metabolism. BMB Rep..

[20] Taskinen M.R., Packard C.J., Boren J. (2019). Emerging Evidence that ApoC-III Inhibitors Provide Novel Options to Reduce the Residual CVD. Curr. Atheroscler. Rep..

[21] Abdul-Wahed A., Guilmeau S., Postic C. (2017). Sweet Sixteenth for ChREBP: Established Roles and Future Goals. Cell Metab..

[22] Kim M.S., Krawczyk S.A., Doridot L., Fowler A.J., Wang J.X., Trauger S.A., Noh H.L., Kang H.J., Meissen J.K., Blatnik M. (2016). ChREBP regulates fructose-induced glucose production independently of insulin signaling. J. Clin. Investig..

[23] Kim M., Astapova I.I., Flier S.N., Hannou S.A., Doridot L., Sargsyan A., Kou H.H., Fowler A.J., Liang G., Herman M.A. (2017). Intestinal, but not hepatic, ChREBP is required for fructose tolerance. JCI Insight.

[24] Haidari M., Leung N., Mahbub F., Uffelman K.D., Kohen-Avramoglu R., Lewis G.F., Adeli K. (2002). Fasting and postprandial overproduction of intestinally derived lipoproteins in an animal model of insulin resistance. Evidence that chronic fructose feeding in the hamster is accompanied by enhanced intestinal de novo lipogenesis and ApoB48-containing lipoprotein overproduction. J. Biol. Chem..

[25] Stanhope K.L. (2016). Sugar consumption, metabolic disease and obesity: The state of the controversy. Crit. Rev. Clin. Lab. Sci..

[26] Sun S.Z., Empie M.W. (2012). Fructose metabolism in humans—What isotopic tracer studies tell us. Nutr. Metab. (Lond.).

[27] Softic S., Cohen D.E., Kahn C.R. (2016). Role of Dietary Fructose and Hepatic De Novo Lipogenesis in Fatty Liver Disease. Dig. Dis. Sci..

[28] Jang C., Hui S., Lu W., Cowan A.J., Morscher R.J., Lee G., Liu W., Tesz G.J., Birnbaum M.J., Rabinowitz J.D. (2018). The Small Intestine Converts Dietary Fructose into Glucose and Organic Acids. Cell Metab..

[29] Gonzalez J.T., Betts J.A. (2018). Dietary Fructose Metabolism By Splanchnic Organs: Size Matters. Cell Metab..

[30] Francey C., Cros J., Rosset R., Creze C., Rey V., Stefanoni N., Schneiter P., Tappy L., Seyssel K. (2019). The extra-splanchnic fructose escape after ingestion of a fructose-glucose drink: An exploratory study in healthy humans using a dual fructose isotope method. Clin. Nutr. ESPEN.

[31] Xiao C., Dash S., Morgantini C., Lewis G.F. (2013). Novel role of enteral monosaccharides in intestinal lipoprotein production in healthy humans. Arterioscler. Thromb. Vasc. Biol..

[32] Taskinen M.R., Soderlund S., Bogl L.H., Hakkarainen A., Matikainen N., Pietilainen K.H., Rasanen S., Lundbom N., Bjornson E., Eliasson B. (2017). Adverse effects of fructose on cardiometabolic risk factors and hepatic lipid metabolism in subjects with abdominal obesity. J. Intern. Med..

[33] Herman M.A., Samuel V.T. (2016). The Sweet Path to Metabolic Demise: Fructose and Lipid Synthesis. Trends Endocrinol. Metab..

[34] Tappy L. (2018). Fructose-containing caloric sweeteners as a cause of obesity and metabolic disorders. J. Exp. Biol..

[35] Spalding K.L., Bernard S., Naslund E., Salehpour M., Possnert G., Appelsved L., Fu K.Y., Alkass K., Druid H., Thorell A. (2017). Impact of fat mass and distribution on lipid turnover in human adipose tissue. Nat. Commun..

[36] Kim S.H., Despres J.P., Koh K.K. (2016). Obesity and cardiovascular disease: Friend or foe?. Eur. Heart J..

[37] Karpe F., Pinnick K.E. (2015). Biology of upper-body and lower-body adipose tissue—Link to whole-body phenotypes. Nat. Rev. Endocrinol..

[38] Schulze M.B. (2019). Metabolic health in normal-weight and obese individuals. Diabetologia.

[39] Neeland I.J., Poirier P., Despres J.P. (2018). Cardiovascular and Metabolic Heterogeneity of Obesity: Clinical Challenges and Implications for Management. Circulation.

[40] Piche M.E., Vasan S.K., Hodson L., Karpe F. (2018). Relevance of human fat distribution on lipid and lipoprotein metabolism and cardiovascular disease risk. Curr. Opin. Lipidol..

[41] Stefan N., Haring H.U., Cusi K. (2019). Non-alcoholic fatty liver disease: Causes, diagnosis, cardiometabolic consequences, and treatment strategies. Lancet Diabetes Endocrinol..

[42] Stefan N., Kantartzis K., Haring H.U. (2008). Causes and metabolic consequences of Fatty liver. Endocr. Rev..

[43] Vernon G., Baranova A., Younossi Z.M. (2011). Systematic review: The epidemiology and natural history of non-alcoholic fatty liver disease and non-alcoholic steatohepatitis in adults. Aliment. Pharmacol. Ther..

[44] Bellentani S., Scaglioni F., Marino M., Bedogni G. (2010). Epidemiology of non-alcoholic fatty liver disease. Dig. Dis..

[45] Estes C., Anstee Q.M., Arias-Loste M.T., Bantel H., Bellentani S., Caballeria J., Colombo M., Craxi A., Crespo J., Day C.P. (2018). Modeling NAFLD disease burden in China, France, Germany, Italy, Japan, Spain, United Kingdom, and United States for the period 2016–2030. J. Hepatol..

[46] Chiu S., Mulligan K., Schwarz J.M. (2018). Dietary carbohydrates and fatty liver disease: De novo lipogenesis. Curr. Opin. Clin. Nutr. Metab. Care.

[47] Sanguesa G., Shaligram S., Akther F., Roglans N., Laguna J.C., Rahimian R., Alegret M. (2017). Type of supplemented simple sugar, not merely calorie intake, determines adverse effects on metabolism and aortic function in female rats. Am. J. Physiol. Heart Circ. Physiol..

[48] Dorn C., Riener M.O., Kirovski G., Saugspier M., Steib K., Weiss T.S., Gabele E., Kristiansen G., Hartmann A., Hellerbrand C. (2010). Expression of fatty acid synthase in nonalcoholic fatty liver disease. Int. J. Clin. Exp. Pathol..

[49] Mitsuyoshi H., Yasui K., Harano Y., Endo M., Tsuji K., Minami M., Itoh Y., Okanoue T., Yoshikawa T. (2009). Analysis of hepatic genes involved in the metabolism of fatty acids and iron in nonalcoholic fatty liver disease. Hepatol. Res..

[50] Paglialunga S., Dehn C.A. (2016). Clinical assessment of hepatic de novo lipogenesis in non-alcoholic fatty liver disease. Lipids Health Dis..

[51] Tappy L., Le K.A. (2010). Metabolic effects of fructose and the worldwide increase in obesity. Physiol. Rev..

[52] Rutledge A.C., Adeli K. (2007). Fructose and the metabolic syndrome: Pathophysiology and molecular mechanisms. Nutr. Rev..

[53] Stanhope K.L., Havel P.J. (2010). Fructose consumption: recent results and their potential implications. Ann. N. Y. Acad. Sci..

[54] Malik V.S., Hu F.B. (2015). Fructose and Cardiometabolic Health: What the Evidence From Sugar-Sweetened Beverages Tells Us. J. Am. Coll. Cardiol..

[55] Fisher F.M., Kim M., Doridot L., Cunniff J.C., Parker T.S., Levine D.M., Hellerstein M.K., Hudgins L.C., Maratos-Flier E., Herman M.A. (2017). A critical role for ChREBP-mediated FGF21 secretion in hepatic fructose metabolism. Mol. Metab..

[56] Solinas G., Boren J., Dulloo A.G. (2015). De novo lipogenesis in metabolic homeostasis: More friend than foe?. Mol. Metab..

[57] Stanhope K.L. (2012). Role of fructose-containing sugars in the epidemics of obesity and metabolic syndrome. Annu. Rev. Med..

[58] Stanhope K.L., Schwarz J.M., Keim N.L., Griffen S.C., Bremer A.A., Graham J.L., Hatcher B., Cox C.L., Dyachenko A., Zhang W. (2009). Consuming fructose-sweetened, not glucose-sweetened, beverages increases visceral adiposity and lipids and decreases insulin sensitivity in overweight/obese humans. J. Clin. Investig..

[59] Faeh D., Minehira K., Schwarz J.M., Periasamy R., Park S., Tappy L. (2005). Effect of fructose overfeeding and fish oil administration on hepatic de novo lipogenesis and insulin sensitivity in healthy men. Diabetes.

[60] Stanhope K.L. (2017). More pieces of the fructose puzzle. J. Intern. Med..

[61] Schwarz J.M., Noworolski S.M., Erkin-Cakmak A., Korn N.J., Wen M.J., Tai V.W., Jones G.M., Palii S.P., Velasco-Alin M., Pan K. (2017). Effects of Dietary Fructose Restriction on Liver Fat, De Novo Lipogenesis, and Insulin Kinetics in Children With Obesity. Gastroenterology.

[62] Vos M.B., Lavine J.E. (2013). Dietary fructose in nonalcoholic fatty liver disease. Hepatology.

[63] Jegatheesan P., De Bandt J.P. (2017). Fructose and NAFLD: The Multifaceted Aspects of Fructose Metabolism. Nutrients.

[64] Jensen T., Abdelmalek M.F., Sullivan S., Nadeau K.J., Green M., Roncal C., Nakagawa T., Kuwabara M., Sato Y., Kang D.H. (2018). Fructose and sugar: A major mediator of non-alcoholic fatty liver disease. J. Hepatol..

[65] Moore J.B. (2019). From sugar to liver fat and public health: Systems biology driven studies in understanding non-alcoholic fatty liver disease pathogenesis. Proc. Nutr. Soc..

[66] Alexander M., Loomis A.K., Fairburn-Beech J., van der Lei J., Duarte-Salles T., Prieto-Alhambra D., Ansell D., Pasqua A., Lapi F., Rijnbeek P. (2018). Real-world data reveal a diagnostic gap in non-alcoholic fatty liver disease. BMC Med..

[67] Lee S.S., Park S.H., Kim H.J., Kim S.Y., Kim M.Y., Kim D.Y., Suh D.J., Kim K.M., Bae M.H., Lee J.Y. (2010). Non-invasive assessment of hepatic steatosis: Prospective comparison of the accuracy of imaging examinations. J. Hepatol..

[68] Reeder S.B., Cruite I., Hamilton G., Sirlin C.B. (2011). Quantitative assessment of liver fat with magnetic resonance imaging and spectroscopy. J. Magn. Reson. Imaging.

[69] Szczepaniak L.S., Nurenberg P., Leonard D., Browning J.D., Reingold J.S., Grundy S., Hobbs H.H., Dobbins R.L. (2005). Magnetic resonance spectroscopy to measure hepatic triglyceride content: Prevalence of hepatic steatosis in the general population. Am. J. Physiol. Endocrinol. Metab..

[70] van de Weijer T., Schrauwen-Hinderling V.B. (2019). Application of Magnetic Resonance Spectroscopy in metabolic research. Biochim. Biophys. Acta Mol. Basis Dis..

[71] European Association for the Study of the Liver, European Association for the Study of Diabetes, European Association for the Study of Obesity (2016). EASL-EASD-EASO Clinical Practice Guidelines for the management of non-alcoholic fatty liver disease. Diabetologia.

[72] Le K.A., Ith M., Kreis R., Faeh D., Bortolotti M., Tran C., Boesch C., Tappy L. (2009). Fructose overconsumption causes dyslipidemia and ectopic lipid deposition in healthy subjects with and without a family history of type 2 diabetes. Am. J. Clin. Nutr..

[73] Sobrecases H., Le K.A., Bortolotti M., Schneiter P., Ith M., Kreis R., Boesch C., Tappy L. (2010). Effects of short-term overfeeding with fructose, fat and fructose plus fat on plasma and hepatic lipids in healthy men. Diabetes Metab..

[74] Lecoultre V., Egli L., Carrel G., Theytaz F., Kreis R., Schneiter P., Boss A., Zwygart K., Le K.A., Bortolotti M. (2013). Effects of fructose and glucose overfeeding on hepatic insulin sensitivity and intrahepatic lipids in healthy humans. Obesity (Silver Spring).

[75] Theytaz F., Noguchi Y., Egli L., Campos V., Buehler T., Hodson L., Patterson B.W., Nishikata N., Kreis R., Mittendorfer B. (2012). Effects of supplementation with essential amino acids on intrahepatic lipid concentrations during fructose overfeeding in humans. Am. J. Clin. Nutr..

[76] Johnston R.D., Stephenson M.C., Crossland H., Cordon S.M., Palcidi E., Cox E.F., Taylor M.A., Aithal G.P., Macdonald I.A. (2013). No difference between high-fructose and high-glucose diets on liver triacylglycerol or biochemistry in healthy overweight men. Gastroenterology.

[77] Surowska A., Jegatheesan P., Campos V., Marques A.S., Egli L., Cros J., Rosset R., Lecoultre V., Kreis R., Boesch C. (2019). Effects of Dietary Protein and Fat Content on Intrahepatocellular and Intramyocellular Lipids during a 6-Day Hypercaloric, High Sucrose Diet: A Randomized Controlled Trial in Normal Weight Healthy Subjects. Nutrients.

[78] Schwarz J.M., Noworolski S.M., Wen M.J., Dyachenko A., Prior J.L., Weinberg M.E., Herraiz L.A., Tai V.W., Bergeron N., Bersot T.P. (2015). Effect of a High-Fructose Weight-Maintaining Diet on Lipogenesis and Liver Fat. J. Clin. Endocrinol. Metab..

[79] Cox C.L., Stanhope K.L., Schwarz J.M., Graham J.L., Hatcher B., Griffen S.C., Bremer A.A., Berglund L., McGahan J.P., Havel P.J. (2012). Consumption of fructose-sweetened beverages for 10 weeks reduces net fat oxidation and energy expenditure in overweight/obese men and women. Eur. J. Clin. Nutr..

[80] Maersk M., Belza A., Stodkilde-Jorgensen H., Ringgaard S., Chabanova E., Thomsen H., Pedersen S.B., Astrup A., Richelsen B. (2012). Sucrose-sweetened beverages increase fat storage in the liver, muscle, and visceral fat depot: A 6-mo randomized intervention study. Am. J. Clin. Nutr..

[81] Silbernagel G., Machann J., Unmuth S., Schick F., Stefan N., Haring H.U., Fritsche A. (2011). Effects of 4-week very-high-fructose/glucose diets on insulin sensitivity, visceral fat and intrahepatic lipids: An exploratory trial. Br. J. Nutr..

[82] Chung M., Ma J., Patel K., Berger S., Lau J., Lichtenstein A.H. (2014). Fructose, high-fructose corn syrup, sucrose, and nonalcoholic fatty liver disease or indexes of liver health: A systematic review and meta-analysis. Am. J. Clin. Nutr..

[83] Chiu S., Sievenpiper J.L., de Souza R.J., Cozma A.I., Mirrahimi A., Carleton A.J., Ha V., Di Buono M., Jenkins A.L., Leiter L.A. (2014). Effect of fructose on markers of non-alcoholic fatty liver disease (NAFLD): a systematic review and meta-analysis of controlled feeding trials. Eur. J. Clin. Nutr..

[84] Ma J., Fox C.S., Jacques P.F., Speliotes E.K., Hoffmann U., Smith C.E., Saltzman E., McKeown N.M. (2015). Sugar-sweetened beverage, diet soda, and fatty liver disease in the Framingham Heart Study cohorts. J. Hepatol..

[85] Sevastianova K., Santos A., Kotronen A., Hakkarainen A., Makkonen J., Silander K., Peltonen M., Romeo S., Lundbom J., Lundbom N. (2012). Effect of short-term carbohydrate overfeeding and long-term weight loss on liver fat in overweight humans. Am. J. Clin. Nutr..

[86] Luukkonen P.K., Sadevirta S., Zhou Y., Kayser B., Ali A., Ahonen L., Lallukka S., Pelloux V., Gaggini M., Jian C. (2018). Saturated Fat Is More Metabolically Harmful for the Human Liver Than Unsaturated Fat or Simple Sugars. Diabetes Care.

[87] Gugliucci A., Lustig R.H., Caccavello R., Erkin-Cakmak A., Noworolski S.M., Tai V.W., Wen M.J., Mulligan K., Schwarz J.M. (2016). Short-term isocaloric fructose restriction lowers apoC-III levels and yields less atherogenic lipoprotein profiles in children with obesity and metabolic syndrome. Atherosclerosis.

[88] Erkin-Cakmak A., Bains Y., Caccavello R., Noworolski S.M., Schwarz J.M., Mulligan K., Lustig R.H., Gugliucci A. (2019). Isocaloric Fructose Restriction Reduces Serum d-Lactate Concentration in Children With Obesity and Metabolic Syndrome. J. Clin. Endocrinol. Metab..

[89] Lee O., Bruce W.R., Dong Q., Bruce J., Mehta R., O’Brien P.J. (2009). Fructose and carbonyl metabolites as endogenous toxins. Chem. Biol. Interact..

[90] Pickens M.K., Yan J.S., Ng R.K., Ogata H., Grenert J.P., Beysen C., Turner S.M., Maher J.J. (2009). Dietary sucrose is essential to the development of liver injury in the methionine-choline-deficient model of steatohepatitis. J. Lipid Res..

[91] Masania J., Malczewska-Malec M., Razny U., Goralska J., Zdzienicka A., Kiec-Wilk B., Gruca A., Stancel-Mozwillo J., Dembinska-Kiec A., Rabbani N. (2016). Dicarbonyl stress in clinical obesity. Glycoconj. J..

[92] Asgari-Taee F., Zerafati-Shoae N., Dehghani M., Sadeghi M., Baradaran H.R., Jazayeri S. (2018). Association of sugar sweetened beverages consumption with non-alcoholic fatty liver disease: A systematic review and meta-analysis. Eur. J. Nutr..

[93] Ter Horst K.W., Serlie M.J. (2017). Fructose Consumption, Lipogenesis, and Non-Alcoholic Fatty Liver Disease. Nutrients.

[94] Kirk E., Reeds D.N., Finck B.N., Mayurranjan S.M., Patterson B.W., Klein S. (2009). Dietary fat and carbohydrates differentially alter insulin sensitivity during caloric restriction. Gastroenterology.

[95] Haufe S., Engeli S., Kast P., Bohnke J., Utz W., Haas V., Hermsdorf M., Mahler A., Wiesner S., Birkenfeld A.L. (2011). Randomized comparison of reduced fat and reduced carbohydrate hypocaloric diets on intrahepatic fat in overweight and obese human subjects. Hepatology.

[96] Perheentupa J., Raivio K. (1967). Fructose-induced hyperuricaemia. Lancet.

[97] Sozen E., Ozer N.K. (2017). Impact of high cholesterol and endoplasmic reticulum stress on metabolic diseases: An updated mini-review. Redox Biol..

[98] Lebeaupin C., Vallee D., Hazari Y., Hetz C., Chevet E., Bailly-Maitre B. (2018). Endoplasmic reticulum stress signalling and the pathogenesis of non-alcoholic fatty liver disease. J. Hepatol..

[99] Henkel A., Green R.M. (2013). The unfolded protein response in fatty liver disease. Semin. Liver Dis..

[100] Lee A.H., Scapa E.F., Cohen D.E., Glimcher L.H. (2008). Regulation of hepatic lipogenesis by the transcription factor XBP1. Science.

[101] Lanaspa M.A., Sanchez-Lozada L.G., Choi Y.J., Cicerchi C., Kanbay M., Roncal-Jimenez C.A., Ishimoto T., Li N., Marek G., Duranay M. (2012). Uric acid induces hepatic steatosis by generation of mitochondrial oxidative stress: Potential role in fructose-dependent and -independent fatty liver. J. Biol. Chem..

[102] Abdelmalek M.F., Lazo M., Horska A., Bonekamp S., Lipkin E.W., Balasubramanyam A., Bantle J.P., Johnson R.J., Diehl A.M., Clark J.M. (2012). Higher dietary fructose is associated with impaired hepatic adenosine triphosphate homeostasis in obese individuals with type 2 diabetes. Hepatology.

[103] Satapati S., Kucejova B., Duarte J.A., Fletcher J.A., Reynolds L., Sunny N.E., He T., Nair L.A., Livingston K.A., Fu X. (2015). Mitochondrial metabolism mediates oxidative stress and inflammation in fatty liver. J. Clin. Investig..

[104] Softic S., Gupta M.K., Wang G.X., Fujisaka S., O’Neill B.T., Rao T.N., Willoughby J., Harbison C., Fitzgerald K., Ilkayeva O. (2017). Divergent effects of glucose and fructose on hepatic lipogenesis and insulin signaling. J. Clin. Investig..

[105] Facchini F., Chen Y.D., Hollenbeck C.B., Reaven G.M. (1991). Relationship between resistance to insulin-mediated glucose uptake, urinary uric acid clearance, and plasma uric acid concentration. JAMA.

[106] Choi H.K., Ford E.S. (2007). Prevalence of the metabolic syndrome in individuals with hyperuricemia. Am. J. Med..

[107] Yu T.Y., Jee J.H., Bae J.C., Jin S.M., Baek J.H., Lee M.K., Kim J.H. (2016). Serum uric acid: A strong and independent predictor of metabolic syndrome after adjusting for body composition. Metabolism.

[108] Lee Y.J., Cho S., Kim S.R. (2014). A possible role of serum uric acid as a marker of metabolic syndrome. Intern. Med. J..

[109] Sun H.L., Pei D., Lue K.H., Chen Y.L. (2015). Uric Acid Levels Can Predict Metabolic Syndrome and Hypertension in Adolescents: A 10-Year Longitudinal Study. PLoS ONE.

[110] Johnson R.J., Nakagawa T., Sanchez-Lozada L.G., Shafiu M., Sundaram S., Le M., Ishimoto T., Sautin Y.Y., Lanaspa M.A. (2013). Sugar, uric acid, and the etiology of diabetes and obesity. Diabetes.

[111] Zurlo A., Veronese N., Giantin V., Maselli M., Zambon S., Maggi S., Musacchio E., Toffanello E.D., Sartori L., Perissinotto E. (2016). High serum uric acid levels increase the risk of metabolic syndrome in elderly women: The PRO.V.A study. Nutr. Metab. Cardiovasc. Dis..

[112] Babio N., Martinez-Gonzalez M.A., Estruch R., Warnberg J., Recondo J., Ortega-Calvo M., Serra-Majem L., Corella D., Fito M., Ros E. (2015). Associations between serum uric acid concentrations and metabolic syndrome and its components in the PREDIMED study. Nutr. Metab. Cardiovasc. Dis..

[113] Yuan H., Yu C., Li X., Sun L., Zhu X., Zhao C., Zhang Z., Yang Z. (2015). Serum Uric Acid Levels and Risk of Metabolic Syndrome: A Dose-Response Meta-Analysis of Prospective Studies. J. Clin. Endocrinol. Metab..

[114] Ouyang X., Cirillo P., Sautin Y., McCall S., Bruchette J.L., Diehl A.M., Johnson R.J., Abdelmalek M.F. (2008). Fructose consumption as a risk factor for non-alcoholic fatty liver disease. J. Hepatol..

[115] Zhang S., Du T., Li M., Lu H., Lin X., Yu X. (2017). Combined effect of obesity and uric acid on nonalcoholic fatty liver disease and hypertriglyceridemia. Medicine (Baltimore).

[116] Liu Z., Que S., Zhou L., Zheng S. (2015). Dose-response Relationship of Serum Uric Acid with Metabolic Syndrome and Non-alcoholic Fatty Liver Disease Incidence: A Meta-analysis of Prospective Studies. Sci. Rep..

[117] Yang C., Yang S., Xu W., Zhang J., Fu W., Feng C. (2017). Association between the hyperuricemia and nonalcoholic fatty liver disease risk in a Chinese population: A retrospective cohort study. PLoS ONE.

[118] Lee J.W., Cho Y.K., Ryan M., Kim H., Lee S.W., Chang E., Joo K.J., Kim J.T., Kim B.S., Sung K.C. (2010). Serum uric Acid as a predictor for the development of nonalcoholic Fatty liver disease in apparently healthy subjects: A 5-year retrospective cohort study. Gut Liver.

[119] Lonardo A., Loria P., Leonardi F., Borsatti A., Neri P., Pulvirenti M., Verrone A.M., Bagni A., Bertolotti M., Ganazzi D. (2002). Fasting insulin and uric acid levels but not indices of iron metabolism are independent predictors of non-alcoholic fatty liver disease. A case-control study. Dig. Liver Dis..

[120] Sirota J.C., McFann K., Targher G., Johnson R.J., Chonchol M., Jalal D.I. (2013). Elevated serum uric acid levels are associated with non-alcoholic fatty liver disease independently of metabolic syndrome features in the United States: Liver ultrasound data from the National Health and Nutrition Examination Survey. Metabolism.

[121] Li Y., Xu C., Yu C., Xu L., Miao M. (2009). Association of serum uric acid level with non-alcoholic fatty liver disease: A cross-sectional study. J. Hepatol..

[122] Xu C., Yu C., Xu L., Miao M., Li Y. (2010). High serum uric acid increases the risk for nonalcoholic Fatty liver disease: A prospective observational study. PLoS ONE.

[123] Choi H.K., Curhan G. (2008). Soft drinks, fructose consumption, and the risk of gout in men: Prospective cohort study. BMJ.

[124] Choi H.K., Willett W., Curhan G. (2010). Fructose-rich beverages and risk of gout in women. JAMA.

[125] Bae J., Chun B.Y., Park P.S., Choi B.Y., Kim M.K., Shin M.H., Lee Y.H., Shin D.H., Kim S.K. (2014). Higher consumption of sugar-sweetened soft drinks increases the risk of hyperuricemia in Korean population: The Korean Multi-Rural Communities Cohort Study. Semin. Arthritis Rheum..

[126] Meneses-Leon J., Denova-Gutierrez E., Castanon-Robles S., Granados-Garcia V., Talavera J.O., Rivera-Paredez B., Huitron-Bravo G.G., Cervantes-Rodriguez M., Quiterio-Trenado M., Rudolph S.E. (2014). Sweetened beverage consumption and the risk of hyperuricemia in Mexican adults: A cross-sectional study. BMC Public Health.

[127] Siqueira J.H., Mill J.G., Velasquez-Melendez G., Moreira A.D., Barreto S.M., Bensenor I.M., Molina M. (2018). Sugar-Sweetened Soft Drinks and Fructose Consumption Are Associated with Hyperuricemia: Cross-Sectional Analysis from the Brazilian Longitudinal Study of Adult Health (ELSA-Brasil). Nutrients.

[128] Nguyen S., Choi H.K., Lustig R.H., Hsu C.Y. (2009). Sugar-sweetened beverages, serum uric acid, and blood pressure in adolescents. J. Pediatr..

[129] Ngo Sock E.T., Le K.A., Ith M., Kreis R., Boesch C., Tappy L. (2010). Effects of a short-term overfeeding with fructose or glucose in healthy young males. Br. J. Nutr..

[130] Cox C.L., Stanhope K.L., Schwarz J.M., Graham J.L., Hatcher B., Griffen S.C., Bremer A.A., Berglund L., McGahan J.P., Keim N.L. (2012). Consumption of fructose- but not glucose-sweetened beverages for 10 weeks increases circulating concentrations of uric acid, retinol binding protein-4, and gamma-glutamyl transferase activity in overweight/obese humans. Nutr. Metab. (Lond.).

[131] Wang D.D., Sievenpiper J.L., de Souza R.J., Chiavaroli L., Ha V., Cozma A.I., Mirrahimi A., Yu M.E., Carleton A.J., Di Buono M. (2012). The effects of fructose intake on serum uric acid vary among controlled dietary trials. J. Nutr..

[132] Tappy L., Morio B., Azzout-Marniche D., Champ M., Gerber M., Houdart S., Mas E., Rizkalla S., Slama G., Mariotti F. (2018). French Recommendations for Sugar Intake in Adults: A Novel Approach Chosen by ANSES. Nutrients.

[133] Caliceti C., Calabria D., Roda A., Cicero A.F.G. (2017). Fructose Intake, Serum Uric Acid, and Cardiometabolic Disorders: A Critical Review. Nutrients.

[134] Kanbay M., Jensen T., Solak Y., Le M., Roncal-Jimenez C., Rivard C., Lanaspa M.A., Nakagawa T., Johnson R.J. (2016). Uric acid in metabolic syndrome: From an innocent bystander to a central player. Eur. J. Intern. Med..

[135] Welsh J.A., Sharma A., Abramson J.L., Vaccarino V., Gillespie C., Vos M.B. (2010). Caloric sweetener consumption and dyslipidemia among US adults. JAMA.

[136] Dhingra R., Sullivan L., Jacques P.F., Wang T.J., Fox C.S., Meigs J.B., D’Agostino R.B., Gaziano J.M., Vasan R.S. (2007). Soft drink consumption and risk of developing cardiometabolic risk factors and the metabolic syndrome in middle-aged adults in the community. Circulation.

[137] Livesey G., Taylor R. (2008). Fructose consumption and consequences for glycation, plasma triacylglycerol, and body weight: Meta-analyses and meta-regression models of intervention studies. Am. J. Clin. Nutr..

[138] Adiels M., Taskinen M.R., Bjornson E., Andersson L., Matikainen N., Soderlund S., Kahri J., Hakkarainen A., Lundbom N., Sihlbom C. (2019). Role of apolipoprotein C-III overproduction in diabetic dyslipidaemia. Diabetes Obes. Metab..

[139] Taskinen M.R., Boren J. (2016). Why Is Apolipoprotein CIII Emerging as a Novel Therapeutic Target to Reduce the Burden of Cardiovascular Disease?. Curr. Atheroscler. Rep..

[140] Borén J., Watts G.F., Adiels M., Söderlund S., Chan D.C., Hakkarainen A., Lundbom N., Matikainen N., Kahri J., Vergès B. (2015). Kinetic and Related Determinants of Plasma Triglyceride Concentration in Abdominal Obesity. Multicenter Tracer Kinetic Study. Arterioscler. Thromb. Vasc. Biol..

[141] Stanhope K.L., Medici V., Bremer A.A., Lee V., Lam H.D., Nunez M.V., Chen G.X., Keim N.L., Havel P.J. (2015). A dose-response study of consuming high-fructose corn syrup-sweetened beverages on lipid/lipoprotein risk factors for cardiovascular disease in young adults. Am. J. Clin. Nutr..

[142] Lambertz J., Weiskirchen S., Landert S., Weiskirchen R. (2017). Fructose: A Dietary Sugar in Crosstalk with Microbiota Contributing to the Development and Progression of Non-Alcoholic Liver Disease. Front. Immunol..

[143] den Besten G., Lange K., Havinga R., van Dijk T.H., Gerding A., van Eunen K., Muller M., Groen A.K., Hooiveld G.J., Bakker B.M. (2013). Gut-derived short-chain fatty acids are vividly assimilated into host carbohydrates and lipids. Am. J. Physiol Gastrointest. Liver Physiol..

[144] Mouzaki M., Comelli E.M., Arendt B.M., Bonengel J., Fung S.K., Fischer S.E., McGilvray I.D., Allard J.P. (2013). Intestinal microbiota in patients with nonalcoholic fatty liver disease. Hepatology.

[145] Jegatheesan P., Beutheu S., Ventura G., Sarfati G., Nubret E., Kapel N., Waligora-Dupriet A.J., Bergheim I., Cynober L., De-Bandt J.P. (2016). Effect of specific amino acids on hepatic lipid metabolism in fructose-induced non-alcoholic fatty liver disease. Clin. Nutr..

[146] Oh J.H., Alexander L.M., Pan M., Schueler K.L., Keller M.P., Attie A.D., Walter J., van Pijkeren J.P. (2019). Dietary Fructose and Microbiota-Derived Short-Chain Fatty Acids Promote Bacteriophage Production in the Gut Symbiont Lactobacillus reuteri. Cell Host Microbe.

[147] Chatterjee A., Duerkop B.A. (2019). Sugar and Fatty Acids Ack-celerate Prophage Induction. Cell Host Microbe.

[148] Mardinoglu A., Wu H., Bjornson E., Zhang C., Hakkarainen A., Rasanen S.M., Lee S., Mancina R.M., Bergentall M., Pietilainen K.H. (2018). An Integrated Understanding of the Rapid Metabolic Benefits of a Carbohydrate-Restricted Diet on Hepatic Steatosis in Humans. Cell Metab..

